# Nuclear size measurement for distinguishing urothelial carcinomas from reactive urothelium on tissue sections

**DOI:** 10.1186/s13000-016-0501-7

**Published:** 2016-06-30

**Authors:** Kate Poropatich, Jason C. Yang, Rajen Goyal, Vamsi Parini, Ximing J. Yang

**Affiliations:** Department of Pathology, Northwestern University Feinberg School of Medicine, 251 E. Huron St., Feinberg 5-705, Chicago, IL 60611 USA; Northwestern University, Evanston, IL USA; Robert H Lurie Comprehensive Cancer Center, Northwestern University Feinberg School of Medicine, Chicago, IL USA

**Keywords:** Nucleomegaly, Reactive urothelial atypia, Urothelial carcinoma in situ (CIS), High-grade urothelial carcinoma (HG UC), Low-grade urothelial carcinoma (LG UC)

## Abstract

**Background:**

Pathological diagnosis of urothelial carcinoma (UC) is primarily based on cytological atypia. It has previously been shown that high-grade (HG) UC, particularly UC in situ cells (CIS), can be over five times the size of a lymphocyte. However, this has not been demonstrated in comparison to reactive urothelium. The objective of this study was to empirically compare the difference in nuclear size of UC cells with reactive urothelial cells.

**Methods:**

Using CellSens imaging software, we measured urothelial nuclear length (*l*) and width (*w*) on digital images of H&E sections. The area (a) of a nucleus was calculated based on the oval shape of most urothelial cells. Lymphocytes were measured to calculate normalized urothelial linear and area ratios.

**Results:**

A total of 1085 urothelial cell nuclei from 60 cases were measured from reactive urothelium, low grade (LG) UC, HG UC and CIS. CIS nuclei were found to have an a 2.75 times larger than reactive nuclei (*p* < 0.001). A nuclear size cut-off of 11 um for *l* and 7 um for *w* was found to be sensitive [98.09 % (95 % CI: 95.60–99.38 %) and 89.31 % (95 % CI: 83.6–91.82 %) for *l* and *w*, respectively] and specific [92.60 % (95 % CI: 87.13–95.82 %) and 85.71 % (95 % CI: 79.49–90.63 %) for *l* and *w*, respectively] for distinguishing CIS from reactive atypia.

**Conclusions:**

Nuclear morphometry can be used to differentiate CIS from reactive atypia. A *l* over 11 um and a *w* over 7 um and is highly sensitive and specific for CIS compared to reactive urothelium. This difference in nuclear size may be used as a tool for differentiating the flat urothelial lesions from reactive urothelium in daily practice.

## Background

Urothelial carcinomas (UC) including papillary tumors and flat urothelial carcinoma in situ (CIS) are diagnosed primarily based on cytological atypia with or without papillary configuation. Much attention has been focused on the morphology of urothelial papillary lesions while less clear morphologic criteria apply to the flat urothelial lesion of CIS and its progression to invasive UC. Urothelial CIS is a particularly important diagnosis because it is the precursor lesion to invasive UC, with high-grade cytologic atypia that may be more pronounced than invasive UC.

One helpful feature for detecting cytologic atypia is nucleomegaly of urothelial cells. Because of the lack of reliable reference urothelial cells in tissue sections, lymphocytes are often used for comparison. It has been proposed that the mean nuclear area of urothelium relative to lymphocytes is the best way to discriminate cytologically malignant urothelium from benign urothelium [1]. CIS nuclei have been shown to be approximately five times larger than a lymphocyte and 1.5 times larger than dysplastic urothelial nuclei whereas reactive urothelial nuclei and lower-grade dysplastic nuclei are approximately twice the size of a lymphocyte [1]. However, it remains unknown what the size difference is to distinguish reactive urothelium from CIS.

Modern technology has afforded use widely available software to practicing pathologists to make objective measurements to quickly differentiate between high-grade dysplasia and reactive atypia. Digital images of the bladder urothelium can be analyzed on high-power to look at a variety of parameters, including nuclear length (*l*), width (*w*) and area (*a*). However, it currently remains unknown what numerical criteria should be applied to distinguish dysplasia from benignancy.

## Methods

**Fig. 1:**
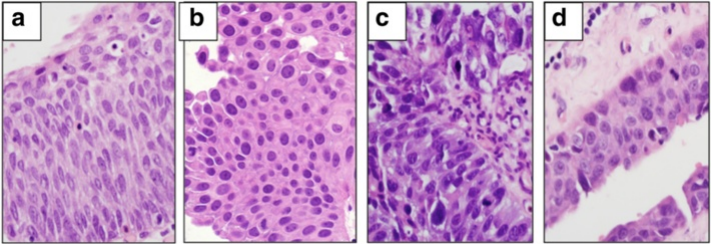
Reactive and Neoplastic Urothelium Categories Photomicrographs Taken With CellSens at 40x. **a** Reactive urothelium **b** LG UC **c** LG UC **d** CIS

The Northwestern Memorial Hospital (Chicago, IL) pathology database was searched for random, nonconsecutive patients over the period of April 2014 to April 2015 who had a biopsy- or surgical-resection diagnosis by a genitourinary specialty pathologist (X.Y.) of reactive urothelium, low grade UC (LG UC) and high grade UC (HG UC) and urothelial CIS (WHO, 2004). Research involving human data was in accordance with the ethical standards of the Northwestern University Institutional Review Board (IRB). Formalin-fixed and paraffin-embedded hematoxylin and eosin (H&E)-stained bladder and ureter biopsies and surgical resection slides were obtained from 60 cases including 10 cases of reactive urothelium, 20 cases of LG UC, 20 cases of HG UC, and 10 cases of urothelial CIS (see Fig. 1a-d).

**Fig. 2:**
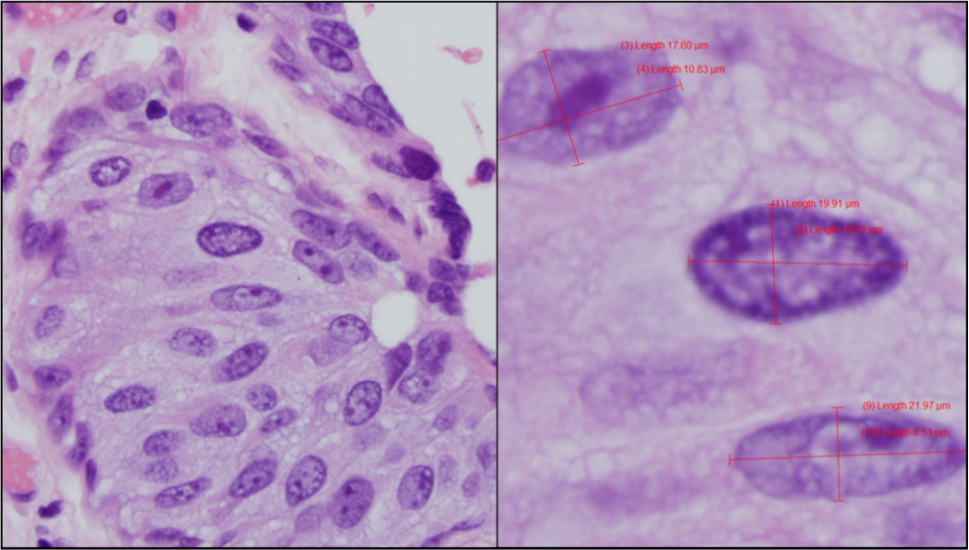
Measuring Nuclei of HGUC and CIS. **a** Example of high-power (40x) photomicrograph of HG UC taken for nuclear size measurements. **b** Representative schematic of CellSens software to measure nuclear *l* and *w*, in a case of urothelial CIS

Using a Nikon digital microscope equipped with a 40x objective and the imaging software *Olympus CellSens* (CellSens,Version 510_UMA_cellSens17-Indus-en_00), *l* and *w* of nuclei were measured on up to five captured images and reviewed blindly by genitourinary pathology specialists (see Fig. 2a-b). For each case, a minimum of ten nuclei per 40x field (0.196 mm^2^) totaling a minimum of 20 nuclei were measured. Among pleomorphic urothelial nuclei, the largest nuclei in each field were selected for measurement. Umbrella cells were excluded from measurement. Lymphocytes on corresponding sections were measured in two dimensions that were then averaged, to be used as the reference standard. Snapshots of each field including a raw version without listed nuclear measurements were saved as *jpeg* files and measurements were extracted into excel for data analysis in order to calculate *a* and size ratios with reference lymphocyte. This was calculated by dividing the average urothelial nuclear *l*, *w* and *a* by the average lymphocyte *l* and *a.*

### Statistical analysis

Statistical significance within each of the urothelial four groups (reactive urothelium, LG UC, HG UC and CIS) was determined by calculation of the standard deviation (SD) from the mean nuclear *l*, *w* and *a* as well as the 95 % confidence interval (CI). Statistically significant differences in nuclear *l*, *w* and *A* between two groups were calculated with a Student’s T test and amongst all groups with a one-way ANOVA. Nuclear size cut-offs for stratification of reactive nuclei from CIS were determined based on calculation of a nuclear *l* and *w* cut-off with a sensitivity and specificity and 95 % CI’s obtained via a ROC-curve analysis. Statistical power was calculated for comparison of reactive nuclei and CIS as a one-tail test with corresponding standard deviations and using an alpha error level of 5 %.

## Results

**Fig. 3:**
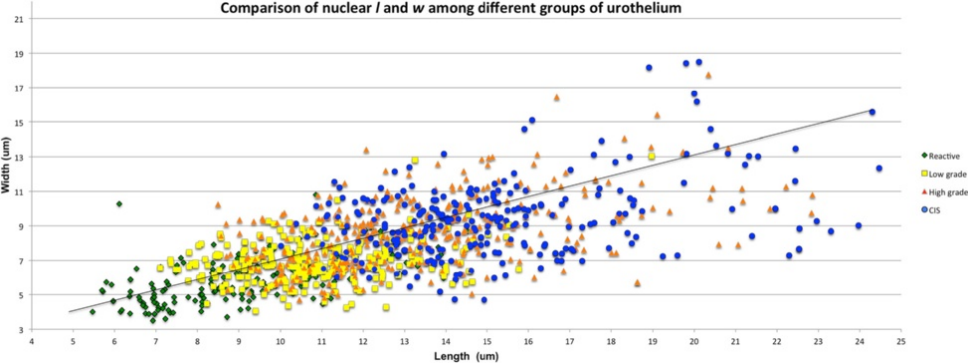
Comparison of Nuclear Length and Width Among Different Groups of Urothelium. Comparison of nuclear *l* and *w* of reactive urothelium (diamond), LG UC (square), HG UC (triangle) and CIS (circle). Trend line shows *w* and *l* increase linearly according to nuclear grade of dysplasia

We measured 1085 nuclei, including 32 from lymphocytes, 168 from reactive urothelium, 271 from LG UC, 384 from HG UC and 262 from urothelial CIS (see Table 1). The mean *l*, *w* and *a* per category increased linearly according to degree of dysplasia and with a tight SD and 95 % CI (see Fig. 3).

**Table 1:** Comparison of Mean Sizes for Nuclei From Reactive Urothelium, LGUC, HGUC and CIS

Cell type	# patients	# nuclei	L um	SD	95 % CI	W um	SD	95 % CI	Area um^2^	SD	95 % CI	Linear Ratio (L_nucleus_)/, (W_nucleus_)/L_lymphocyte_^a^	Area ratio (A_nucleus_/A_lymphocyte_)^b^
Reactive Urothelium	10	168	8.91	1.95	[8.62, 9.2]	5.75	1.29	[5.55, 5.95]	42.03	16.50	[39.5, 44.5]	1.85, 1.2	2.32
LG UCa	20	271	10.85	1.82	[10.63, 11.07]	6.92	1.20	[6.78, 7.06]	60.46	17.68	[58.36, 62.56]	2.26, 1.44	3.33
HG UCa	20	384	13.56	3.52	[13.21, 13.91]	8.82	2.54	[8.57, 9.07]	100.24	68.15	[93.42, 107.06]	2.82, 1.83	5.53
CIS	10	262	15.17	3.03	[14.8, 15.54]	9.35	2.22	[9.08, 9.62]	115.5	45.3	[110.01, 120.99]	3.15, 1.94	6.37

*LG UC* low-grade urothelial carcinoma, *HG UC* high-grade urothelial carcinoma, *CIS* carcinoma in situ, *SD* standard deviation, *CI* confidence interval, *l* length, *w* width, *a* area

^a^Mean *l* and *w* (um) of lymphocyte = 4.81

^b^Mean *a* (um^2^) of lymphocyte = 18.12

**Fig. 4:**
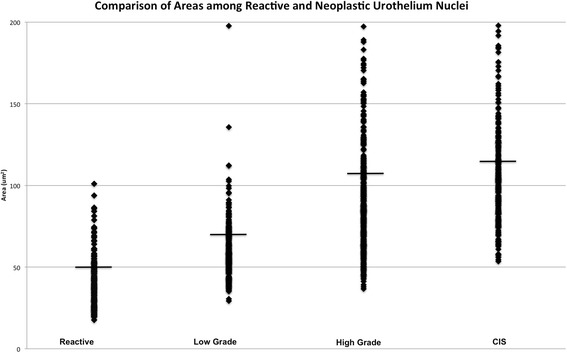
Comparison of Areas Among Reactive and Neoplastic Nuclei. Comparison of nuclear *a* for reactive urothelial, LG UC, HG UC and CIS
III. Nuclear size stratification system to differentiate reactive urothelium from CIS
  Of most clinical interest is distinguishing CIS from reactive atypia, for which in our case the differences in *l* and *w* between these two groups each had statistical powers of 100 %. Based on the largest size differentials in *l, w* and *a* between reactive urothelium and CIS, nuclear cut off points were devised to separate approximately 95 % of CIS nuclei from reactive urothelium nuclei. A nuclear size cut-off of 11 um for *l* and 7 um for *w* was found to be sensitive [98.09 % (95 % CI: 95.60–99.38 %) and 89.31 % (95 % CI: 83.6–91.82 %) for *l* and *w*, respectively] and specific [92.60 % (95 % CI: 87.13–95.82 %) and 85.71 % (95 % CI: 79.49–90.63 %) for *l* and *w*, respectively] for distinguishing CIS from reactive atypia. Similarly, a nuclear *a* of 65.45 um^2^ stratifies 95 % of CIS nuclei from reactive urothelium nuclei. For this reason, we prefer a nuclear *l* and *w* stratification system of 11 um and 7 um, respectively, for reliably detecting CIS and minimizing the likelihood that a reactive urothelial cell is mistaken as CIS (see Fig. 5). The sensitivity of using this length cut off for *l* is 98.09 %, 95 % CI [0.84–0.92]. The specificity of this *l* cut-off is 0.92, 95 % CI [0.87–0.92] and *w* cut-off is 0.86, 95 % CI [0.79, 0.90].

I. Differences in nuclear length and width
  Using ANOVA for intergroup comparisons, all categories were statistically significant from one another for *l* and *w* measurement comparisons. The mean nuclear *l* for each category was measured as follows: reactive urothelium, 8.91 um [SD: 1.95, 95 % CI (8.62, 9.2)]; LG UC, 10.85 um [SD: 1.82, 95 % CI (10.63, 11.07)], HG UC, 13.56 um [SD: 3.52, 95 % CI (13.21, 13.91)]; CIS, 15.17 um [SD: 3.03, 95 % CI (14.8, 15.54)]. In comparison to the reference lymphocyte, which was circular-shaped and had both an average *l* and *w* of 4.81 um, nuclei from HG UC and CIS had *l* size ratios as follows: reactive urothelium, 1.85 (*P* < 0.001); LG UC, 2.26 longer (*P* < 0.001); HG UC, 2.82 times longer (*P* < 0.001); CIS, 3.15 (*P* < 0.001). Similarly, the mean nuclear *w* increased linearly according to nuclear grade as follows: reactive urothelium, 5.75 um [SD: 1.29, 95 % CI (5.55, 5.95)]; LG UC, 6.92 [SD: 1.2, 95 % CI (6.78, 7.06)]; HG UC, 8.92 [SD: 2.54, 95 % CI (8.57, 9.07)]; CIS, 9.35 um [SD: 2.22, 95 % CI (9.08, 9.62)]. Size ratios for nuclear *w* were as follows: reactive urothelium, 1.2 (*P* < 0.001); LG UC, 1.44 (*P* < 0.001); HG UC, 1.83 (*P* < 0.001); CIS, 1.94 (*P* < 0.001).
  The greatest difference in nuclear *l* and *w* were consistently observed between reactive urothelium nuclei and CIS nuclei—CIS nuclei were 1.70 times longer and 1.55 times wider than nuclei of reactive urothelium. This difference was less for dysplastic nuclei of UC compared to reactive lymphocytes. Measured nuclei of HG UC were only1.52 times longer and 1.53 times wider than measured nuclei of reactive urothelium. HG UC had the greatest *w* compared to all other categories, and this was over twice as large as the *w* for LG UC. CIS nuclei were both longer and wider than low and high-grade dysplastic nuclei of UC.
II. Differences in nuclear area
  Similar to nuclear *l* and *w*, the *a* of LG UC, HG UC and CIS nuclei increased linearly in comparison to both the reference lymphocyte and reactive urothelial nuclei. However, sizes were polarized based on group—LG UC had an *a* closer in value to reactive urothelium whereas HG UC and CIS nuclei were of comparable sizes (see Fig. 4). The measured *a* were as follows: reactive urothelium, 42.03 um^2^ [SD: 16.5, 95 % CI (39.5, 44.5)]; LG UC, 60.46 um^2^ [SD: 17.68, 95 % CI (58.36, 62.56)]; HG UC, 100.24 um^2^ [SD: 68.15, 95 % CI (93.42, 107.06)]; CIS, 115.5 um^2^ [SD: 115.5, 95 % CI (110.01, 120.99)]. Size ratios with the reference lymphocyte were greatest for HG UC and CIS nuclei and were calculated for all groups as follows: reactive urothelium, 2.32 (*P* < 0.001); LG UC, 3.33 (*P* < 0.001); HG UC, 5.53 (*P* < 0.001); CIS, 6.37 times larger (*P* < 0.001). Similarly, nuclei of HG UC and CIS had the greatest difference in *a* when compared with reactive urothelium and were 2.75 um^2^ and 2.38 um^2^ times larger (*P* < 0.001). This size difference was considerably smaller (1.43 um^2^) when comparing LG UC with reactive urothelium (*P* < 0.001).

**Fig. 5:**
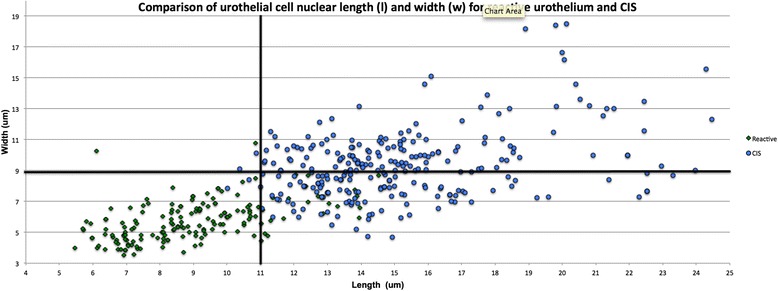
Comparison of Nuclear Cell Length and Width for Reactive Urothelium and CIS. A cut-off nuclear *l* of 11 um and nuclear *w* of 7 um (solid lines) differentiates CIS nuclei (circles) from reactive urothelial nuclei (diamonds)

## Discussion

In this study, we have shown using the nuclear length and width on a conventional digital camera can be very helpful to distinguish benign and high grade urothelial carcinoma, particularly useful for differentiating the diagnostically challenging cases of reactive atypical urothelium from CIS. Perhaps one of the most diagnostically challenging dilemmas is distinguishing CIS from reactive urothelial aytpia when no other urothelial abnormalities are present. While stage is the most important prognostic indicator of long-term prognosis, grade is important at the time of biopsy of urothelial lesions for determining risk of progression and association with invasive disease^2^. According to the TNM (tumor, node, metastasis) staging system, high-grade cytology is seen in 90 % of superficial urothelial neoplasms (Ta and T1) with invasion confined to the lamina propria [2].

In normal urothelium, there are basal, intermediate and superficial umbrella cells that span three to seven layers based on the state of bladder distension [2]. Compared to the hyperchromatic nuclei of the basal cells, the intermediate cells have round-to-oval shaped nuclei that often include grooves and have clear or amphophilic cytomplasm. Overlying these intermediate cells is the superficial layer coined umbrella cells, which have variably sized nuclei and voluminous eosinophilic cytoplasm. Urothelium may show varying degrees of architectural distortion and loss of polarity of these cell layers without becoming dysplastic [2].

Flat urothelial lesions of CIS are hallmarked by the partial or full-thickness involvement of the urothelium by cytologically atypical cells. It can diffusely involve the surface of the bladder and is usually treated intravesically with Bacillus Calmette-Guerin (BCG). In other clinical scenarios, pagetoid CIS may only present as a few neoplastic cells with enlarged nuclei scattered throughout an otherwise orderly arranged urothelium [2]. While nucleomegaly is one of the key features of cytologic atypical in UC, it can also be seen in reactive urothelium [3–6]. It is prudent to first analyze urothelium at the10x objective lens because CIS can typically be distinguished from reactive urothelium based on the presence of pleomorphic cells with enlarged, hyperchromatic nuclei compared to the monotonous enlarged nuclei of reactive urothelium [3]. Confirmation at a higher-power field can be made in relation to adjacent lymphocytes. CIS nuclei more commonly show central clearing with eccentrically-placed nucleoli whereas nuclei of reactive urothelium have vesicular chromatin with centrally-placed nucleoli [1, 3, 5, 7, 8]. Both entities may have mitotic figures, however in benign reactive conditions these are more commonly located in the rapidly dividing basal cell layer. In some cases, the morphologic differentiation between reactive atypia and dysplasia is subtle with the only difference being the degree of nuclear enlargement.

The diagnosis of invasive UC is based on histologic evidence of invasion of neoplastic cells into the underlying lamina propria. According to the World Health Organization (WHO) 2003/International Society of Urological Pathologists [WHO(2003)/ISUP] classification, invasive UC is graded according to a two-tier system defined as high-grade (HG) and low-grade (LG) [3]. LG UC is less common and may be seen in the nested UC variant where it can be overlooked due to its deceptively bland cytology [2]. Cytologic features of LG UC include more subtle variations in nuclear size, shape and chromatin texture and loss of nuclear polarity [2]. While infrequent and typically located in the lower-half of the urothelium, mitotic figures may also be seen at higher levels of the urothelium. In comparison, features that characterize HG UC include disorganized epithelium with more frequent mitoses, clumped chromatin, prominent nucleoli and importantly, nucleomegaly.

To date, this is the first study to systematically evaluate the specific difference in nuclear size amongst categories of benign and dysplastic urothelium in a large cohort of patients. We determined ratios for nuclear size in reference to the *l* and *w* of lymphocytes as a way to standardize the data (see Fig. 5) and to determine whether a size criteria can be applied to differentiate CIS from reactive atypia. We show that nuclei that are over 11 um and 7 um in *l* and *w,* respectively, are most likely CIS in the absence of a HG infiltrative lesion. This size cut-off was determined with simple microscopy tools available that are in most clinical settings. Using this size cut-off to diagnose CIS has a sensitivity of 98.09 and 89.31 % for *l* and *w* respectively, with fairly high specificities of 86.90 and 85.71 % for *l* and *w,* respectively. While the optimal size criteria would have higher specificities without lowering the sensitivity, in this case choosing a higher specificity would substantially decrease the sensitivity and likely result in a risky number of false negative CIS cases. While the risk of false positives with this case cannot be excluded based on the considerably higher *l* sensitivity compared to specificity, it is important to remember that these cut-offs are first and foremost devised as a screening technique for CIS, particularly in the case of small biopsies where erring on the conservative side could result in long-term consequences of high-grade malignancy.

We found that among CIS nuclei (*n* = 262) selected from resection and TURBT specimens, CIS nuclei have a mean area that exceeds previous estimates and were approximately 6.37 times larger than a lymphocyte (*P* < 0.001). Additionally, we found that CIS nuclei are approximately 2.75 times larger in *a* compared to reactive urothelium. We also show that CIS is a lesion with even greater nucleomegaly compared to its invasive LG and HG UC counterparts when using a *l*, *w,* and *a* nuclear size comparison. CIS nuclei are closer in size to HG UC nuclei versus LG UC and reactive urothelium nuclei in both *l* and *w*. This confirms that LG UC is a lesion of low cytologic atypia when using nucleomegaly as a sole measuring criterion and that LG UC is difficult to distinguish from reactive urothelial atypia because of their morphologic resemblance.

Some of the limitations of this study include the difficulty in distinguishing LG UC from reactive urothelial cell when papillary architecture commonly seen in LG UC is absent. Additional limitations include not accounting for whether CIS lesions analyzed in this study are part of a HG UC process that was not picked up on biopsy specimens. Additionally, this study could benefit from the inclusion of UC cases arising outside the bladder or ureter, such as the renal pelvis.

By accounting for cases of CIS in both the urinary bladder and ureters, it is the intent of this study to present practicing pathologists with a simple size-criterion to diagnose what is otherwise thought to be a widely underdiagnosed entity in the common scenarios of focal CIS involvement and thinned-out urothelium that presents as ‘clinging’ CIS.

## Conclusions

In this study we found that nuclear morphometry can be used to differentiate CIS from reactive atypia—a particularly challenging clinical scenario that can present in time-sensitive instances where nuclear features are often the only sign of atypia, such as at frozen section for establishing clean margins for bladder resection specimens. More specifically, by analyzing 1085 nuclei including from lymphocytes, reactive urothelium, LG UC, HG UC and urothelial CIS, we find that the mean *l*, *w* and *a* per category increased linearly according to degree of dysplasia and with a tight SD and 95 % CI. For the first time we establish stringent criteria and guidelines for differentiating reactive urothelial nuclei from those of urothelial CIS with high specificity and sensitivity: a *l* over 11 um and a *w* over 7 um are accurate cut-offs for distinguishing the former from the latter. This difference in nuclear size may be used as a tool for differentiating the flat urothelial lesions from reactive urothelium in daily practice.

## Abbreviations

*a*, area; CI, confidence interval; CIS, carcinoma in situ; HG UC, high-grade urothelial carcinoma (HG UC); *l*, length; LG UC, low-grade urothelial carcinoma (LG UC); SD, standard deviation; *w*, width.
